# Multiplexed detection of nuclear immediate early gene expression reveals hippocampal neuronal subpopulations that engage in the acquisition and updating of spatial experience

**DOI:** 10.3389/fnint.2025.1660536

**Published:** 2025-12-03

**Authors:** Thu-Huong Hoang, Denise Manahan-Vaughan

**Affiliations:** Department of Neurophysiology, Medical Faculty, Ruhr University Bochum, Bochum, Germany

**Keywords:** rodents, spatial updating, fluorescence *in situ* hybridization, immediate early genes, hippocampus

## Abstract

Acquired spatial representations are not static. Each re-exposure to the spatial environment stimulates retrieval of the stored experience followed by information re-encoding, including updating if the environment has changed. It remains unclear if the same neurons are involved in these three events. Here, we used a multiplexed fluorescence *in situ* hybridization (FISH) approach that detected “time-locked” nuclear immediate early gene (IEG) expression to identify hippocampal neuronal ensembles that were engaged in the acquisition of a spatial representation, as well as its subsequent stabilization and/or updating. Responses were assessed in distal CA1 (dCA1) and proximal CA1 (pCA1) of the dorsal hippocampus of male rats. Homer1a was used to detect neuronal recruitment triggered by novel learning of a holeboard environment (HB). cFos and Arc expression were used to detect ensemble stability and/or expansion, or ensemble remodeling, respectively, that was triggered by animal exposure to the now familiar HB that included novel objects (HBO) 25 min after the initial HB exposure. Novel HB exposure resulted in nuclear Homer1a expression in both dCA1 and pCA1. Subsequent HBO triggered significant cFos and Arc expression only in dCA1. IEG co-labeling (Homer1a/cFos, Homer1a/Arc and Homer1a/cFos/Arc) was also only evident in dCA1, reflecting both re-iteration and remodeling of dCA1, but not pCA1 ensembles.

In sum, we show that the contiguous acquisition and updating of spatial representations recruits distinct populations of CA1-neurons reflecting ensemble selection and stabilization, as well as ensemble remodeling. Moreover, whereas dCA1 and pCA1 are involved in the acquisition of the original spatial representation, only dCA1 is engaged in representation updating related to changes in spatial content information.

## 1 Introduction

The acquisition of the initial scaffold of a spatial and/or associative experience can occur in matter of seconds (Piette et al., 2020). However, the acquisition of more detailed spatial representation and/or its updating, is a process that requires time spent in, and movement through, the environment, re-exposure to the same or the updated environment, as well as perception of the saliency and valence of spatial details (Caragea and Manahan-Vaughan, 2021; Griesbauer et al., 2022; Nyberg et al., 2022; Tse et al., 2023). In rats, this process of context- and experience-dependent spatial information acquisition and adaptation is reflected by the stabilization, rate remapping and global remapping of hippocampal place fields (Colgin, 2020) and in the enablement of hippocampal long-term potentiation (LTP) and long-term depression (LTD) by the learning and/or updating of specific components of a spatial representation (Hagena and Manahan-Vaughan, 2024).

Although neuronal ensembles have been reported following one-trial aversive learning in rodents (Kupke and Oliveira, 2025), the reactivation of which leads to reinstatement behavioral indicators of memory retrieval (Vasudevan et al., 2024), and neuronal populations that express Arc or cFos have been identified during non-aversive learning conducted in mice (Kwapis et al., 2019; Autore et al., 2023; Chen et al., 2022), little is known about how neuronal ensembles contribute to non-aversive forms of spatial learning in rats. Moreover, it has been proposed that rather than have designated neuronal populations that retain specific elements of a spatial representation, the hippocampus utilizes manifold representations of similar (Stacho and Manahan-Vaughan, 2022) or different spatial experiences (Fenton, 2024) that allows the disambiguation, storage and retrieval of context-dependent space. Here, fluorescence *in situ* hybridization (FISH), used in a multiplexed approach, to detect experience dependent nuclear expression of different immediate early genes triggered by novel learning and information updating, may help resolve some of these controversies.

Subfields of the hippocampus show specialization for the storage, disambiguation and retrieval of different aspects of spatial experience. It has been controversially discussed that different subfields of the cornus ammonis (CA) region of the hippocampus proper, as well as the dentate gyrus, support pattern completion and pattern separation (Kesner, 2013; Quian Quiroga, 2020; Suthana et al., 2021; Yassa and Stark, 2011). However, evidence also exists that the dentate gyrus and proximal CA1 region (pCA1) support pattern separation, whereas the distal CA1 region (dCA1) supports pattern completion (Lee et al., 2020). It has also been proposed that information about non-spatial (e.g., item) identity (“what”) and allocentric space (“where”) is delivered to the hippocampus by an “offshoot” of the dorsal and ventral visual streams (de Haan and Cowey, 2011), whereby spatial information is delivered by temporoammonic afferents to pCA1 from the medial entorhinal cortex (EC) and non-spatial information is transferred to dCA1 by lateral EC afferents, resulting in the corresponding functional compartmentalization of the response of dCA1 and pCA1 to spatial and non-spatial experience (Allison et al., 2023; Vandrey et al., 2021). This interplay was confirmed in studies where nuclear expression of immediate early genes (IEG) was used to study how neurons of dCA1 and pCA1 are engaged in registering novel spatial information: whereas an overt spatial change triggered nuclear IEG expression in both dCA1 and pCA1, the inclusion of novel items into a known spatial environment triggered IEG expression in dCA1 (Hoang et al., 2018).

In the present study, our goal was to examine whether differentiated nuclear expression of IEGs resulting from the initial exposure of rats to an overt change in the spatial environment (introduction of a novel holeboard), followed by insertion of novel physical objects into the holeboard holes, can reveal information encoding and updating dynamics in the dorsal CA1 region of the hippocampus. In particular, we examined the extent to which the same neurons are engaged in information acquisition and updating of a representation of the same spatial environment. Our strategy was based on the latency required for specific IEGs to reach peak nuclear expression after a behavioral learning event (Guzowski et al., 1999; Minatohara et al., 2015). We used FISH to detect neuronal Homer1a expression as a biomarker of novel exposure of adult rats to a holeboard. This IEG reaches peak nuclear expression 30–40 min after a specific experience (Bottai et al., 2002; Hoang et al., 2018, 2021; Hoang and Manahan-Vaughan, 2024; Vazdarjanova et al., 2002). To identify neurons that were engaged in subsequent information encoding and/or updating we used FISH to detect nuclear expression of cFos and the activity-regulated cytoskeleton-associated protein (Arc), both of which show peak nuclear expression 5–6 min after a specific experience (Guzowski et al., 1999; Hoang et al., 2018; Saidov et al., 2018; Vazdarjanova et al., 2002). Whereas Homer1a and cFos are transcription factors, Arc is a cytosolic-associated protein (Lyford et al., 1995; Yelhekar et al., 2024). Homer1a plays a role in rendering excitatory synapses amenable for synaptic plasticity (Clifton et al., 2019) and nuclear expression of Homer1a in the hippocampus is increased by induction of long-term potentiation (LTP) and long-term depression (LTD) (Hoang et al., 2021), cellular mechanisms that support spatial learning (Hagena and Manahan-Vaughan, 2024). Associative learning experiences trigger expression of cFos, whereby most studies have examined the role of cFos-expressing neuronal ensembles in the acquisition and retrieval of fear memory (Minatohara et al., 2015). A role for Arc in restructuring dendrites and dendritic spines has also been described (Chowdhury et al., 2006; Peebles et al., 2010; Mikuni et al., 2013; Okuno et al., 2012, 2018). We thus, used Homer1a as a biomarker of information encoding resulting in the recruitment of neurons into an ensemble, whereas cFos and Arc indicated whether these neurons were re-engaged during information updating following object insertion into the holeboard, or whether new neurons were recruited into the ensemble. Our findings reveal the dynamic nature of ongoing spatial information encoding and indicate that this process is likely to comprise both the strengthening and weakening of hippocampal neuronal networks.

## 2 Materials and methods

### 2.1 Animals

**FIGURE 1:**
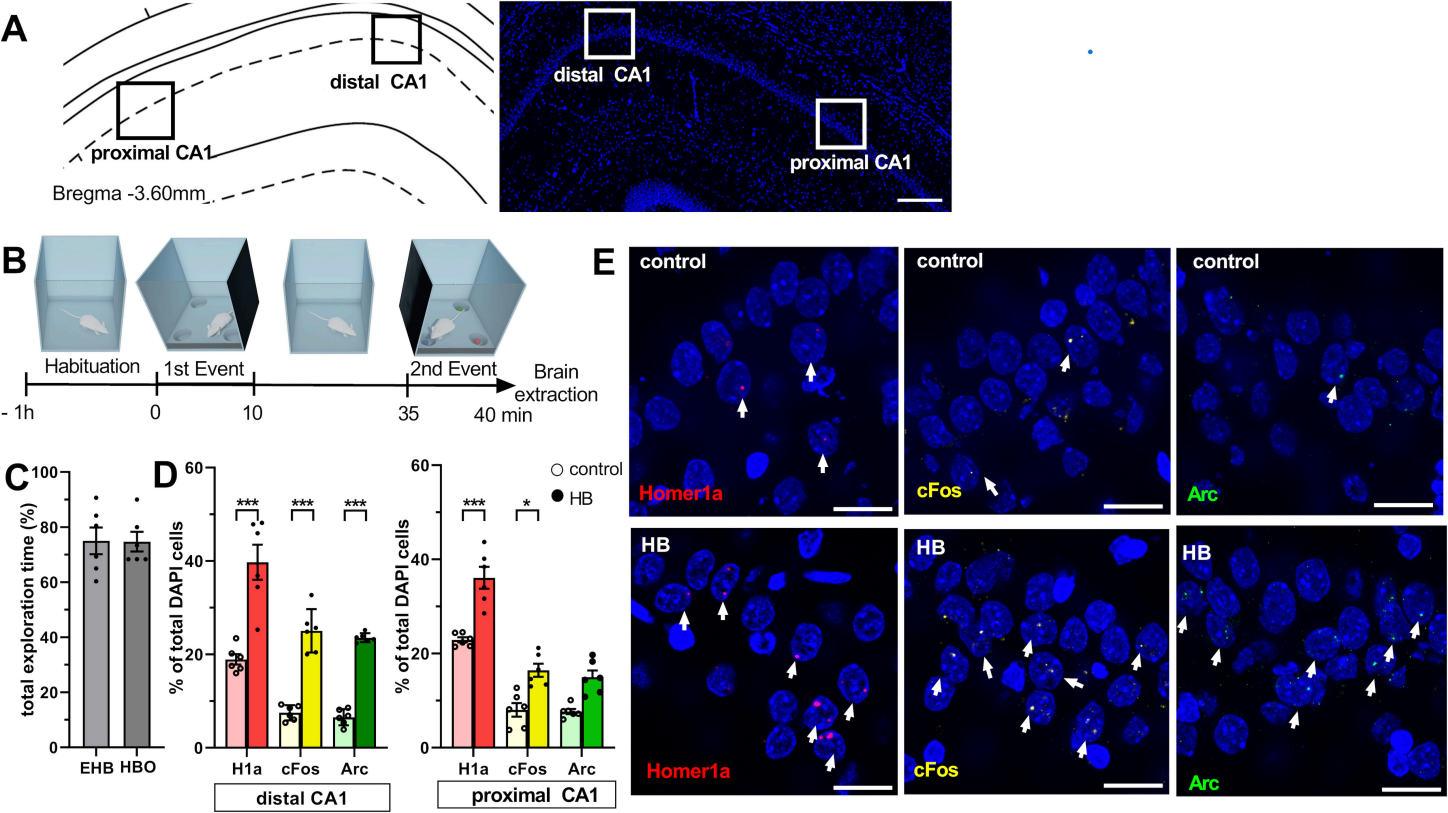
Novel spatial experience significantly increases nuclear expression of Homer1a, Arc and cFos in the CA1 region. **(A)** Definition of regions of interest in the CA1 region (left). Image (right) shows a DAPI-stained section at the level of CA1 region of the dorsal hippocampus [3.6 mm posterior from Bregma as determined by a rat brain atlas (Paxinos and Watson, 2014)]. Z-stacks were obtained in the distal and proximal subcompartments of the CA1 region (black and white squares). Scale bar: 200 μm. **(B)** Experimental design: After having resided in the same experiment room overnight, animals underwent 1 h-habituation in the test chamber on the day of the experiment. During the first event, an empty holeboard was introduced to the animal in the chamber. After 10 min exploration time, the holeboard was removed. Animals rested in the chamber for 25 min. Then, the same holeboard was re-inserted to the recording box, but this time, it contained 3 small objects that were placed inside 3 of the 4 holeboard holes. After 5 min of exploration, brain extraction occurred. **(C)** Bar chart shows the percentage (mean ± SEM) of total exploration time for the exposure to empty holeboard (EHB) during the first holeboard exposure event, and holeboard with objects (HBO) during the 2nd exposure event. During each exposure event, animals exhibited a high exploration percentage. **(D)** Bar charts show the relative percentage (mean ± SEM) of nuclear Homer1a (red bars), cFos (yellow bars) and Arc (green bars) mRNA expression in neuronal nuclei of the distal (left) and proximal CA1 (right). Novel holeboard (HB) exposure led to a significant increase in Homer1a expression in both distal and proximal CA1 compared to controls (no exploration event). Re-exposure to the holeboard containing novel objects (HBO) significantly triggered cFos expression in the distal and proximal CA1 compared to controls. Significant increase in nuclear Arc expression was only evident in the distal but not the proximal CA1 after HBO (***p < 0.001, *p < 0.05). **(E)** Representative images of nuclear Homer1a, cFos and Arc mRNA expression in the distal CA1 of a rat that underwent exploration tasks (HB, bottom row) and in a control rat (control, no exploration event, upper row). Nuclear Homer1a, cFos and Arc mRNA signals are indicated by red, yellow and green dots, respectively. Nuclei were counterstained with DAPI. White arrows indicate IEG positive nuclei. Images were acquired using a wide-field fluorescence microscope at the final magnification of 63×. Scale bars: 20 μm.

The study was conducted in accordance with the European Communities Council Directive of September 22nd, 2010 (2010/63/EU) for care of laboratory animals. The experiments were approved in advance by the local state authority [Landesamt für Verbraucherschutz und Ernährung (LAVE), North Rhein-Westfalia]. All efforts were made to minimize the number of animals used for this study, specifically by conducting power calculations to establish the minimal cohort size for meaningful statistical analyses. The animals also served as their own controls. They were housed in sibling groups in a temperature and humidity-controlled vivarium (Scantainer Ventilated Cabinets, Scanbur A/S, Denmark) with a constant 12-h light–dark cycle (lights on from 7 a.m. to 7 p.m.), controlled temperature (22°C ± 2°C) and humidity (55% ± 5%). Food and water were available *ad libitum* throughout all experiments. In total, 24 male Wistar rats (7–8-weeks old) were used for this study. Female rats were not used because adult female rats become stressed by the presence of male rats (DeBold and Miczek, 1981) and this could alter the outcome of IEG expression and data interpretation. This was also part of our strategy to keep animal numbers to a minimum (reduction of variability of responses) and minimize stressed-related elevations of IEG in the hippocampus (Figure 1A; see, also, comments on FISH strategy, below).

### 2.2 Behavioral experiments

The behavioral habituation and learning protocols were conducted as previously described (Hoang et al., 2018). Animals were handled by the experimenter for 15 min per day for a minimum of 5 days. Then they were habituated to the experiment room and the test chambers for 1 h per day, for 3 consecutive days prior to the commencement of the experiments. The test chambers were 40 × 40 × 40 cm in size, open at the top, and were made of gray washable acrylic panels (Perspex^®^, polymethylmethacrylate, PMMA). The chamber interior was accessible via a translucent PMMA front wall that was held in place by means of tracks installed at the ends of the abutting chamber walls. The front wall could be moved upward by sliding it along these tracks. After the final habituation day, animals remained in the same experiment room overnight. On the day of the first experiment, the animals were placed in the chamber for 1 h before the acquisition phase commenced. The front wall of the chamber was moved upward so that a holeboard (HB) could be inserted gently into the chamber. The HB was 39.8 × 39.8 × 39.5 cm in proportions (gray PMMA) and included four holes (with a closed base) that were 5.5 cm in diameter and 5 cm deep and were placed equidistantly 2 cm from the edges of HB (Figure 1B).

### 2.3 Acquisition-reexposure experiment

Behavioral learning events comprised two separate events: The first event comprised exposure to the novel empty HB. Here, the front panel of the chamber was moved upward so that the HB could be inserted. The animals hopped onto the HB as it was moved into the chamber until one side touched the back wall. The HB was left in place for 10 min and then removed (Figure 1B). After HB removal, the animals typically moved to a corner of their chamber and closed their eyes or rested with eyes open.

The second event comprised the re-introduction of the, now familiar, HB into the chamber, whereby this time it included three novel objects (4 × 2 × 2 cm) placed (one each) into three of the four HB holes (HBO) (Figure 1B). The total exposure time to HBO was 5 min. Events were timed so that HB exposure was aligned with the subsequent detection of peak nuclear Homer1a expression (Hoang et al., 2018) and HBO exposure was aligned with peak expression of Arc (Hoang et al., 2018) and cFos (see below). For this, HB and HBO exposure were separated by 25 min (i.e., 10 min HB exposure plus 25 min pause), meaning that HBO began 35 min after commencing HB. In the interval between HB and HBO, animals resided undisturbed in the recording chamber. The objects used for HBO were distinct from one another and did not extend above the surface of the holeboard. The animals approached the holes, inserted their noses inside the holes to examine the objects and sometimes lifted them out of the holes with both paws. Their typical behavior was to return the objects to the same holes if they had lifted them outside the holes. The configuration of the objects was randomly assigned for each rat.

Immediately after the conclusion of HBO (40 min after starting HB exposure) brains were rapidly removed in a smooth movement that involved taking the animals out of the chamber and placing them in a guillotine. This movement took no longer than 3 s. Brains were immediately shock frozen in iso-pentane (placed in a small metal container and maintained at a temperature of −60°C), surrounded by dry ice, and then stored at −80°C until further processing. An aged-matched control group of male rats (*n* = 6) was included in the study. These animals underwent the same handling and habituation procedures as the test animals. On the day of the experiment, they resided undisturbed in the chamber for at least 1 h. No learning events were implemented. Their brains were quickly removed and flash frozen using the same timeline as described above.

### 2.4 Acquisition-only experiment

In this set of experiments, animals explored a novel holeboard containing novel small items (HBOa) for 10 min, as described above (Supplementary Figure 1A). Then the holeboard was removed from the recording chamber. Animals resided in the chamber for further 30 min. Brain removal occurred 40 min after the start of the exploration. In this experiment, HBOa was the only behavioral condition experienced. Homer1a was used as a biomarker to detect neurons activated by HB, or HBOa, whereas Arc and cFos served as biomarkers to detect neurons that were activated by HBO 40 min after the start of novel HB exposure.

Holeboard environment, HBOa and HBO exploration were video-monitored, and the experiment was discontinued, or the data discarded, if an animal spent less than 5 min exploring the empty HB, HBOa or less than 3 min exploring during HBO.

The exploration time was recorded when the animals first started moving after HB insertion and used their noses to explore the environment. Later, the percentage of exploration time was calculated as exploration time divided by the total exposure time to the HB, HBO or HBOa and multiplied by 100 (Figure 1C, Supplementary Figure 1B).

### 2.5 Multiplexed fluorescence *in situ* hybridization

Under RNAse free conditions, brains were sectioned using a cryostat (Leica CM 3050S, Leica Biosystem GmbH, Wetzlar, Germany). Coronal slices (20 μm) containing hippocampus (from ca. 3.0 to 4.0 mm posterior from Bregma) were collected, mounted directly on slides (superfrost plus^®^ Gerhard Menzel GmbH, Braunschweig, Germany) and stored at −80°C until further processing.

Activity-regulated cytoskeleton-associated protein, Homer1a and cFos cDNA plasmids were prepared commercially (Genscript Biotech, Piscataway Township, New Jersey, USA) using transcripts described by Lyford et al. (1995), Brakeman et al. (1997) and Saidov et al. (2018). The cRNA probes were prepared using a transcription kit (Invitrogen Ambion Maxiscript Kit, ThermoFischer Scientific Waltham, USA) and a premixed RNA labeling mix containing Digoxigenin-11-UTP (Roche Diagnostics, Basel, Switzerland) or Fluorescein-12-UTP (Roche Diagnostics, Basel, Switzerland) or Biotin-16-UTP (Roche Diagnostics, Basel, Switzerland). For this study, Arc RNA was labeled with Biotin, Homer1a RNA with Fluorescein and cFos RNA with Digoxigenin. The generated RNA probes were purified using an RNA CleanUp Kit (Monarch^®^RNA Cleanup Kit, New England BioLabs, Ipswich, USA). Yield and integrity were verified using gel electrophoresis and the concentration was measured by using a QuantiFluor^®^RNA system (Promega, Madison, USA).

From each animal, we chose 3 consecutive dorsal hippocampal brain sections (ca. 3.6 mm posterior to Bregma) and left the slides at room temperature (RT) until they were defrosted. Later, slides were fixed for 10 min in ice-cold 4% paraformaldehyde in fresh filtered phosphate buffered saline, quickly washed in 2-fold concentrate saline-sodium citrate (2xSSC) buffer (RNAse free), incubated in acetic anhydride solution and briefly washed in 2xSSC (RNAse free) at RT. Then, the slides underwent a prehybridization process for 15 min in a mixture of 4xSSC and Formamide (1:1) (RNAse free) at 37°C. The mixture of labeled RNA probes (Arc-Biotin, Homer1a-Fluorescein and cFos-Digoxigenin) was diluted with a concentration of 1 g/1 ul in 1× hybridization buffer (Sigma-Aldrich, St. Louis, USA), treated at 90°C for 5 min and then quickly put on ice. After the prehybridization, the slides were incubated with the hybridization buffer containing RNA probes for the hybridization process in a humid chamber that contained filter paper soaked with 2xSSC/50 deionized Formamide (Sigma-Aldrich, St. Louis, USA) in a dilution of 1:1. The hybridization process in the humid chamber lasted ca. 17 h at 56 °C.

Additionally, a negative control, FISH test, was included to verify the specificity of the hybridized signal. For this, no RNA probes were added to this brain slide (data not shown). After the hybridization, slides underwent stringent washing steps: thrice in 2xSSC at 56°C, then in 2xSSC containing RNAse A at 37°C, again in 2xSSC at 37°C, twice in 0.5xSSC at 56°C, 0.5xSSC at RT, twice in 1xSSC at RT and finally thrice in tris-buffered saline (TBS) at RT. Slides were then treated for 15 min with 3% H_2_O_2_ solution at RT in order to block the endogenous peroxidase.

The Arc-Biotin protocol was previously established (Hoang et al., 2018). Arc-Biotin signal detection was conducted using the following steps:

1. 70 min incubation in TBS-Tween (0.05%, Polysorbate 20) containing 1% Bovine Serum Albumin (BSA) and 20% Streptavidin (Vectorlabs, SP2002, Burlingame, USA).
2. 90 min incubation in TBS-Tween (0.05%) containing 1% BSA and 20% Biotin (Vectorlabs, SP2002, Burlingame, USA) and streptavidin-peroxidase (1:2000, Jackson Immuno Research, Scottsdale, Arizona, USA).
3. Washed 4 times in TBS (5 min each).
4. Incubation for 20 min in TBS containing 1% biotinylated tyramine and 0.01% H_2_O_2_.
5. Washed 4 times in TBS (5 min each) and 60 min incubation in TBS-Tween (0.05%) containing streptavidin CF488 (Biotium, Biotrend GmbH, Cologne, Germany).
6. Sections were left in TBS at 4°C and protected from light overnight until the next detection.

On the next day, the Homer-Fluorescein signal was detected. The protocol to detect the Homer1a-Fluorescein signal was previously established (Hoang et al., 2018) and comprised the following:

1. Incubation for 70 min in TBS-Tween (0.05%, Polysorbate 20) containing 10% n-Goat serum (Histoprime, Biozol, Hamburg, Germany) and 20% Streptavidin (Vectorlabs, SP2002).
2. 90 min incubation in TBS-Tween (0.05%) containing 1% n-Goat serum (Histoprime, Biozol, Hamburg, Germany) and 20% Biotin (Vectorlabs, SP2002, Burlingame, USA) and anti-fluorescein-peroxidase (1:2000, Jackson Immuno Research, Scottsdale, Arizona, USA).
3. Washed 4 times (5 min each) in TBS.
4. Incubation for 20 min in TBS containing 1% biotinylated tyramine and 0.01% H_2_O_2_.
5. Washed 4 times (5 min each) in TBS and 60 min incubation in TBS-Tween (0.05%) containing 1% n-Goat serum and streptavidin Cy5 (Jackson Immuno Research, Scottsdale, Arizona, USA).
6. Sections were left in TBS at 4°C and kept away from light overnight until the next detection.

On the next day, the cFos-Digoxigenin signal was detected as follows:

1. Incubation for 70 min in TBS-Tween (0.05%, Polysorbate 20) containing 10% n-Goat serum (Histoprime, Biozol, Hamburg, Germany) and 20% Streptavidin (Vectorlabs, SP2002, Burlingame, USA).
2. Incubation for 90 min in TBS-Tween (0.05%) containing 1% n-Goat serum (Histoprime, Biozol, Hamburg, Germany) and 20% Biotin (Vectorlabs, SP2002, Burlingame, USA) and anti-digoxigenin-peroxidase (1:2000, Jackson Immuno Research, Scottsdale, Arizona, USA).
3. Washed 4 times (5 min each) in TBS.
4. Incubation for 20 min in TBS containing 1% biotinylated tyramine and 0.01% H_2_O_2_.
5. Washed 4 times (5 min each) in TBS followed by 60 min incubation in TBS-Tween (0.05%) containing 1% n-Goat serum and streptavidinCy7 (Invitrogen, Thermo Fischer Scientific, Waltham, Massachusetts, USA).

Later, slides were washed 4 times (5 min each) in TBS, rinsed in double-distilled water, quickly dipped in 70% ethanol and finally stained using 1% Sudan Black B (Merck KGaA, Sigma-Aldrich, St. Louis, USA) in 70% ethanol (Oliveira et al., 2010). Finally, slides were rinsed in distilled water, air dried and mounted in antifading mounting medium (immunoSelect^®^, Dianova, Hamburg, Germany) containing 4′-6-diamidino-2-phenylindole (DAPI).

Peak nuclear Homer 1a expression occurs 30–40 min after a specific induction event (Bottai et al., 2002). Peak nuclear Arc and cFos expression occur 5–6 min and 6–8 min after a specific induction event, respectively (Saidov et al., 2018; Vazdarjanova et al., 2002). Afterwards, the IEG diffuses into the cytoplasm (Bottai et al., 2002; Saidov et al., 2018; Vazdarjanova et al., 2002). For this reason, we assume that if we detect cytoplasmic expression of a given IEG at the time-point of nuclear detection, this is an indicator that the animals underwent another salient experience in the time before the experiment was started. Given that the animals resided in their homecages before and after HB/HBO exposure, this salient experience would have to be stress-related. This can be caused by unexpected noises (in the room or corridor outside the room), strangers entering the room, the presence of adult male rodents (for females), or females in oestrus (for males), or stress-related (22 Hz) vocalizations by animals in the proximity of the room (Cullinan et al., 1995; Inagaki and Ushida, 2017; Manahan-Vaughan, 2017, 2018; Schreiber et al., 1991). For this reason, we ensured that the animals were well-habituated to handling by the experimenter, that only male rats were used and that experiments were conducted under quiet and calm conditions.

### 2.6 Data analysis

We focused our analysis on the dorsal CA1 region, given that this subregion of the hippocampus expresses long-term potentiation (LTP) when novel HB exposure is coupled with afferent stimulation, whereas long-term depression (LTD) is facilitated by novel HBO exposure coupled with afferent stimulation (Kemp and Manahan-Vaughan, 2004). Moreover, novel HB and novel HBO exposure results in a distinct pattern of nuclear IEG expression in the distal and proximal parts of the dorsal CA1 region (Hoang et al., 2018). In other words, the CA1 generates distinct functionally relevant “encoding” responses to novel HB and novel HBO exposure.

Z-stacks were obtained in the distal CA1 (dCA1) and proximal CA1 (pCA1) at a 63× magnification using a widefield fluoresence microscope (Zeiss Apotome, Oberkochen, Germany) (Figure 1A). Region of interest (ROI) that contained dCA1 and pCA1 were determined based on their anatomical locations (Naber et al., 2001) and comprised a standardized area of 150 μm × 200 μm (Figure 1A). Three consecutive slices of each animal were used for the analysis, whereby we analyzed both hemispheres of each slice and calculated the mean of these three slices. Complete DAPI stained nuclei that showed no evidence of cutting on the edges (of the slides) either in the x, y or z planes, were marked using Fiji software (Schindelin et al., 2012). Neurons were distinguished from glial cells and endothelial cells on the basis of cell morphology and size (Félétou, 2011).

Neurons were checked for nuclear mRNA expression of Homer1a, Arc and cFos that peaked in the nuclei of the CA1 neurons, in an experimenter-blind manner. Based on the fluorescent label, these were detected as red punctae for Homer1a, green punctae for Arc and yellow punctae for cFos (Figure 1E). Percentages of IEG-mRNA positive cells were calculated per total counted neurons for each subregion of each rat. The designation “positive nuclei” was given to cells that contained intense intranuclear foci of IEG-mRNA fluorescent signals (Figure 1D). Nuclei that did not contain any intranuclear foci representing a fluorescent signal of IEG-mRNA were counted as negative. The total number of cells analyzed for each ROI of each slide of each animal was on average 80. Then, the relative percentage of single labeled IEG-mRNA positive cells was calculated relative to the total DAPI counts for each ROI. Additionally, to examine the contribution of the co-labeling of IEGs within the activated neurons, the number of neurons that expressed only Homer1a (H1a-only) or cFos (cFos-only) or Arc (Arc-only), were compared with co-labeling of IEG-mRNA. Here, percentages were calculated by dividing labeled counts of IEGs and total IEG positive counts. Final results are presented as average means of percentages ± standard error of the mean (SEM) for each group. Graphs were generated using GraphPrism (GraphPad Software Version 8, San Diego, California USA). Venn diagrams were created in Python 3.11.8 using a custom script created with the matplotlib_venn package.

### 2.7 Statistical analysis

Control and test animals comprised *n* = 6 each. All values were verified for normal distribution using the Kolmogorov-Smirnow test with Lilliefors correction. Statistical analysis was performed using Statistica software (version 14.0.1.25, TIBCO Software Inc., Santa Clara, CA, USA). Multifactorial analysis of variance (mANOVA) was performed, followed by a subsequent Tukey HSD *post-hoc* test for pairwise comparison between factor groups or regions or IEGs. For multiple comparisons between co-labeled factors, Fisher’s LSD *post-hoc* test was performed and summarized in Tables 1, 2. The significance level was set at *p* < 0.05.

**TABLE 1:** Summary of responses to novel holeboard exposure followed by exposure to the familiar holeboard containing novel items.

	Summary	*P*-value
dH1a-only:control vs. pH1a-only:control	ns	0.7741
dH1a-only:control vs. dcFos-only:control	****	**<0.0001**
dH1a-only:control vs. dArc-only:control	****	**<0.0001**
dH1a-only:control vs. d(H1a+cFos)-only:control	****	**<0.0001**
dH1a-only:control vs. d(H1a+Arc)-only:control	****	**<0.0001**
dH1a-only:control vs. d(H1a+Arc+cFos):control	****	**<0.0001**
dcFos-only:control vs. pcFos-only:control	ns	0.1388
dcFos-only:control vs. dArc-only:control	ns	0.4092
dcFos-only:control vs. d(H1a+cFos)-only:control	**	**0.0017**
dcFos-only:control vs. d(H1a+Arc)-only:control	***	**0.0003**
dcFos-only:control vs. d(Arc+cFos)-only:control	****	**<0.0001**
dcFos-only:control vs. d(H1a+Arc+cFos):control	****	**<0.0001**
dArc-only:control vs. pArc-only:control	ns	0.8639
dArc-only:control vs. d(H1a+cFos)-only:control	*	**0.0191**
dArc-only:control vs. d(H1a+Arc)-only:control	**	**0.0052**
dArc-only:control vs. d(Arc+cFos)-only:control	**	**0.0052**
dArc-only:control vs. d(H1a+Arc+cFos):control	***	**<0.0001**
d(H1a+cFos)-only:control vs. d(H1a+Arc)-only:control	ns	0.6410
d(H1a+cFos)-only:control vs. d(Arc+cFos)-only:control	*	**0.0157**
d(H1a+cFos)-only:control vs. d(H1a+Arc+cFos):control	**	**0.0083**
d(H1a+cFos)-only:control vs. p(H1a+cFos)-only:control	ns	0.3489
d(H1a+Arc)-only:control vs. d(Arc+cFos)-only:control	*	**0.0499**
d(H1a+Arc)-only:control vs. d(H1a+Arc+cFos):control	*	**0.0289**
d(H1a+Arc)-only:control vs. p(H1a+Arc)-only:control	ns	0.3489
d(Arc+cFos)-only:control vs. d(H1a+Arc+cFos):control	ns	0.8183
d(Arc+cFos)-only:control vs. p(Arc+cFos)-only:control	ns	0.8469
d(H1a+Arc+cFos):control vs. p(H1a+Arc+cFos):control	ns	0.8777
pH1a-only:control vs. pcFos-only:control	****	**<0.0001**
pH1a-only:control vs. pArc-only:control	ns	0.6252
pH1a-only:control vs. p(H1a+cFos)-only:control	****	**<0.0001**
pH1a-only:control vs. p(H1a+Arc)-only:control	****	**<0.0001**
pH1a-only:control vs. p(H1a+Arc+cFos):control	****	**<0.0001**
pcFos-only:control vs. p(H1a+cFos)-only:control	ns	0.4436
pcFos-only:control vs. p(H1a+Arc)-only:control	ns	0.0689
pcFos-only:control vs. p(Arc+cFos)-only:control	****	**<0.0001**
pcFos-only:control vs. p(H1a+Arc+cFos):control	****	**<0.0001**
pArc-only:control vs. p(H1a+cFos)-only:control	ns	0.2106
pArc-only:control vs. p(H1a+Arc)-only:control	*	**0.0217**
pArc-only:control vs. p(Arc+cFos)-only:control	****	**<0.0001**
pArc-only:control vs. p(H1a+Arc+cFos):control	****	**<0.0001**
p(H1a+cFos)-only:control vs. p(H1a+Arc)-only:control	ns	0.2888
p(H1a+cFos)-only:control vs. p(Arc+cFos)-only:control	***	**0.0004**
p(H1a+cFos)-only:control vs. p(H1a+Arc+cFos):control	***	**0.0007**
p(H1a+Arc)-only:control vs. p(Arc+cFos)-only:control	***	**0.0004**
p(H1a+Arc)-only:control vs. p(H1a+Arc+cFos):control	*	**0.0179**
p(Arc+cFos)-only:control vs. p(H1a+Arc+cFos):control	ns	0.9607
d(H1a+cFos)-only:control vs. p(H1a+cFos)-only:control	ns	0.2110
d(H1a+Arc)-only:control vs. p(H1a+Arc)-only:control	ns	0.6472
d(Arc+cFos)-only:control vs. p(Arc+cFos)-only:control	ns	0.7959
dH1a-only:control vs. dH1a-only:HB	****	**<0.0001**
dcFos-only:control vs. dcFos-only:HB	*	**0.0384**
dArc-only:control vs. dArc-only:HB	ns	0.3687
d(H1a+Arc)-only:control vs. d(H1a+Arc)-only:HB	*	0.0482
d(Arc+cFos)-only:control vs. d(Arc+cFos)-only:HB	*	0.0178
d(H1a+cFos)-only:control vs. d(H1a+cFos)-only:HB	****	**<0.0001**
d(H1a+Arc+cFos):control vs. d(H1a+Arc+cFos):HB	****	**<0.0001**
pH1a-only:control vs. pH1a-only:HB	*	**0.0214**
pcFos-only:control vs. pcFos-only:HB	ns	0.3403
pArc-only:control vs. pArc-only:HB	ns	0.4078
p(H1a+cFos)-only:control vs. p(H1a+cFos)-only:HB	*	**0.0397**
p(H1a+Arc)-only:control vs. p(H1a+Arc)-only:HB	ns	0.3708
p(Arc+cFos)-only:control vs. p(Arc+cFos)-only:HB	ns	0.3071
p(H1a+Arc+cFos):control vs. p(H1a+Arc+cFos):HB	ns	0.2928
dH1a-only:HB vs. pH1a-only:HB	****	**<0.0001**
dH1a-only:HB vs. dcFos-only:HB	****	**<0.0001**
dH1a-only:HB vs. dArc-only:HB	****	**<0.0001**
dH1a-only:HB vs. d(H1a+cFos)-only:HB	****	**<0.0001**
dH1a-only:HB vs. d(H1a+Arc)-only:HB	****	**<0.0001**
dH1a-only:HB vs. d(H1a+Arc+cFos):HB	****	**<0.0001**
dcFos-only:HB vs. pcFos-only:HB	ns	0.7224
dcFos-only:HB vs. dArc-only:HB	ns	0.7191
dcFos-only:HB vs. d(H1a+cFos)-only:HB	ns	0.0521
dcFos-only:HB vs. d(Arc+cFos)-only:HB	ns	0.0826
dcFos-only:HB vs. d(H1a+Arc+cFos):HB	ns	0.7296
dArc-only:HB vs. pArc-only:HB	ns	0.9202
dArc-only:HB vs. d(H1a+cFos)-only:HB	ns	0.1122
dArc-only:HB vs. d(H1a+Arc)-only:HB	ns	0.6607
dArc-only:HB vs. d(Arc+cFos)-only:HB	*	**0.0368**
dArc-only:HB vs. d(H1a+Arc+cFos):HB	ns	0.9889
d(H1a+cFos)-only:HB vs. d(H1a+Arc)-only:HB	*	**0.0434**
d(H1a+cFos)-only:HB vs. d(Arc+cFos)-only:HB	***	**0.0003**
d(H1a+cFos)-only:HB vs. d(H1a+Arc+cFos):HB	ns	0.1092
d(H1a+Arc)-only:HB vs. d(Arc+cFos)-only:HB	ns	0.0974
d(H1a+Arc)-only:HB vs. d(H1a+Arc+cFos):HB	ns	0.6708
d(Arc+cFos)-only:HB vs. d(H1a+Arc+cFos):HB	**	**0.0041**
pH1a-only:HB vs. pcFos-only:HB	****	**<0.0001**
pH1a-only:HB vs. pArc-only:HB	****	**<0.0001**
pH1a-only:HB vs. p(H1a+cFos)-only:HB	****	**<0.0001**
pH1a-only:HB vs. p(H1a+Arc)-only:HB	****	**<0.0001**
pH1a-only:HB vs. p(H1a+Arc+cFos):HB	****	**<0.0001**
pcFos-only:HB vs. pArc-only:HB	ns	0.5389
pcFos-only:HB vs. p(H1a+cFos)-only:HB	*	**0.0251**
pcFos-only:HB vs. p(H1a+Arc)-only:HB	ns	0.9828
pcFos-only:HB vs. p(Arc+cFos)-only:HB	*	**0.0194**
pcFos-only:HB vs. p(H1a+Arc+cFos):HB	*	**0.0283**
pArc-only:HB vs. p(H1a+cFos)-only:HB	ns	0.1015
pArc-only:HB vs. p(H1a+Arc)-only:HB	ns	0.5532
pArc-only:HB vs. p(Arc+cFos)-only:HB	**	**0.0034**
pArc-only:HB vs. p(H1a+Arc+cFos):HB	**	**0.0053**
p(H1a+cFos)-only:HB vs. p(H1a+Arc)-only:HB	*	**0.0265**
p(H1a+cFos)-only:HB vs. p(Arc+cFos)-only:HB	****	**<0.0001**
p(H1a+cFos)-only:HB vs. p(H1a+Arc+cFos):HB	****	**<0.0001**
p(H1a+Arc)-only:HB vs. p(Arc+cFos)-only:HB	*	**0.0184**
p(H1a+Arc)-only:HB vs. p(H1a+Arc+cFos):HB	*	**0.0265**
p(Arc+cFos)-only:HB vs. p(H1a+Arc+cFos):HB	ns	0.8823
d(H1a+Arc+cFos):HB vs. p(H1a+Arc+cFos):HB	**	**0.004**
d(H1a+cFos)-only:HB vs. p(H1a+cFos)-only:HB	ns	0.9597
d(H1a+Arc)-only:HB vs. p(H1a+Arc)-only:HB	ns	0.7992
d(Arc+cFos)-only:HB vs. p(Arc+cFos)-only:HB	ns	0.3326

The table describes the outcome of Fisher’s multiple comparisons of immediate early gene (IEG)-positive cells in the distal CA1 (dCA1) and proximal CA1 (pCA1) regions of the hippocampus. Nuclear expression of Arc, cFos and Homer1a (H1a) (*n* = 6) were compared to expression in control hippocampus (*n* = 6). In the table “only” signifies that cells were assessed that expressed only one or two of the three IEGs, a plus sign (+) indicates that double or triple labeling of nuclei was assessed. H1a expression was used to determine the effects of novel holeboard (HB) exposure. Both Arc and cFos expression were used to detect the effect of subsequent exposure to the same holeboard that now contained novel objects (HBO). The summary column reports the significance levels (*p < 0.05,

**p < 0.01,

***p < 0.001,

****p < 0.0001) and indicates if effects were not significant (ns). The *P*-value column reports *p*-values, whereby significant effects are highlighted in bold font. dArc, distal CA1 Arc; dcFos, distal CA1 cFos; dH1a, distal CA1 Homer1a; pArc, proximal CA1 Arc; pcFos, proximal CA1 cFos; pH1a, proximal CA1 Homer1a; control, group of animals that did not undergo learning task; HB, group of animals that were exposed to a novel holeboard and 25 min later were exposed to the same holeboard containing novel items in the holeboard hole; ns, not significant.

**TABLE 2:** Summary of responses to novel holeboard containing novel items.

	Summary	*P*-value
dH1a-only:control vs. pH1a-only:control	ns	0.3431
dH1a-only:control vs. dcFos-only:control	****	**<0.0001**
dH1a-only:control vs. dArc-only:control	****	**<0.0001**
dH1a-only:control vs. d(H1a+cFos)-only:control	****	**<0.0001**
dH1a-only:control vs. d(H1a+Arc)-only:control	****	**<0.0001**
dH1a-only:control vs. d(H1a+Arc+cFos):control	****	**<0.0001**
dcFos-only:control vs. pcFos-only:control	ns	0.8334
dcFos-only:control vs. dArc-only:control	ns	0.8860
dcFos-only:control vs. d(H1a+cFos)-only:control	ns	0.1444
dcFos-only:control vs. d(H1a+Arc)-only:control	*	0.032
dcFos-only:control vs. d(Arc+cFos)-only:control	ns	0.7743
dcFos-only:control vs. d(H1a+Arc+cFos):control	ns	0.7430
dArc-only:control vs. pArc-only:control	ns	0.2043
dArc-only:control vs. d(H1a+cFos)-only:control	ns	0.1093
dArc-only:control vs. d(H1a+Arc)-only:control	*	**0.0224**
dArc-only:control vs. d(Arc+cFos)-only:control	ns	0.8860
dArc-only:control vs. d(H1a+Arc+cFos):control	ns	0.6376
d(H1a+cFos)-only:control vs. d(H1a+Arc)-only:control	ns	0.4866
d(H1a+cFos)-only:control vs. d(Arc+cFos)-only:control	ns	0.0814
d(H1a+cFos)-only:control vs. d(H1a+Arc+cFos):control	ns	0.2565
d(H1a+cFos)-only:control vs. p(H1a+cFos)-only:control	ns	0.9411
d(H1a+Arc)-only:control vs. d(Arc+cFos)-only:control	*	**0.0154**
d(H1a+Arc)-only:control vs. d(H1a+Arc+cFos):control	ns	0.0683
d(H1a+Arc)-only:control vs. p(H1a+Arc)-only:control	ns	0.3503
d(Arc+cFos)-only:control vs. d(H1a+Arc+cFos):control	ns	0.5390
d(Arc+cFos)-only:control vs. p(Arc+cFos)-only:control	ns	0.7743
d(H1a+Arc+cFos):control vs. p(H1a+Arc+cFos):control	ns	0.8345
pH1a-only:control vs. pcFos-only:control	****	**<0.0001**
pH1a-only:control vs. pArc-only:control	ns	0.3594
pH1a-only:control vs. p(H1a+cFos)-only:control	****	**<0.0001**
pH1a-only:control vs. p(H1a+Arc)-only:control	****	**<0.0001**
pH1a-only:control vs. p(H1a+Arc+cFos):control	****	**<0.0001**
pcFos-only:control vs. p(H1a+cFos)-only:control	ns	0.2387
pcFos-only:control vs. p(H1a+Arc)-only:control	ns	0.3105
pcFos-only:control vs. p(Arc+cFos)-only:control	ns	0.8334
pcFos-only:control vs. p(H1a+Arc+cFos):control	ns	0.7440
pArc-only:control vs. p(H1a+Arc)-only:control	ns	0.9217
pArc-only:control vs. p(Arc+cFos)-only:control	ns	0.2604
pArc-only:control vs. p(H1a+Arc+cFos):control	ns	0.5547
p(H1a+cFos)-only:control vs. p(H1a+Arc)-only:control	ns	0.8688
p(H1a+cFos)-only:control vs. p(Arc+cFos)-only:control	ns	0.1656
p(H1a+cFos)-only:control vs. p(H1a+Arc+cFos):control	ns	0.3934
p(H1a+Arc)-only:control vs. p(Arc+cFos)-only:control	ns	0.2214
p(H1a+Arc)-only:control vs. p(H1a+Arc+cFos):control	ns	0.4909
p(Arc+cFos)-only:control vs. p(H1a+Arc+cFos):control	ns	0.5916
d(H1a+cFos)-only:control vs. p(H1a+cFos)-only:control	ns	0.9111
d(H1a+Arc)-only:control vs. p(H1a+Arc)-only:control	ns	0.3504
d(Arc+cFos)-only:control vs. p(Arc+cFos)-only:control	ns	0.7743
dH1a-only:control vs. dH1a-only:HBOa	ns	0.8985
dcFos-only:control vs. dcFos-only:HBOa	ns	0.9919
dArc-only:control vs. dArc-only:HBOa	ns	0.9679
d(H1a+Arc)-only:control vs. d(H1a+Arc)-only:HBOa	ns	0.6168
d(Arc+cFos)-only:control vs. d(Arc+cFos)-only:HBOa	ns	1.000
d(H1a+cFos)-only:control vs. d(H1a+cFos)-only:HBOa	ns	0.7588
d(H1a+Arc+cFos):control vs. d(H1a+Arc+cFos):HBOa	ns	0.9242
pH1a-only:control vs. pH1a-only:HBOa	ns	0.5894
pcFos-only:control vs. pcFos-only:HBOa	ns	0.7840
pArc-only:control vs. pArc-only:HBOa	ns	0.1993
p(H1a+cFos)-only:control vs. p(H1a+cFos)-only:HBOa	ns	0.1155
p(H1a+Arc)-only:control vs. p(H1a+Arc)-only:HBOa	ns	0.7967
p(Arc+cFos)-only:control vs. p(Arc+cFos)-only:HBOa	ns	0.7743
p(H1a+Arc+cFos):control vs. p(H1a+Arc+cFos):HBOa	ns	0.6189
dH1a-only:HBOa vs. pH1a-only:HBOa	ns	0.1075
dH1a-only:HBOa vs. dcFos-only:HBOa	****	**<0.0001**
dH1a-only:HBOa vs. dArc-only:HBOa	****	**<0.0001**
dH1a-only:HBOa vs. d(H1a+cFos)-only:HBOa	****	**<0.0001**
dH1a-only:HBOa vs. d(H1a+Arc)-only:HBOa	****	**<0.0001**
dH1a-only:HBOa vs. d(H1a+Arc+cFos):HBOa	****	**<0.0001**
dcFos-only:HBOa vs. pcFos-only:HBOa	ns	0.6354
dcFos-only:HBOa vs. dArc-only:HBOa	**ns**	**0.8464**
dcFos-only:HBOa vs. d(H1a+cFos)-only:HBOa	ns	0.0797
dcFos-only:HBOa vs. d(Arc+cFos)-only:HBOa	ns	0.7666
dcFos-only:HBOa vs. d(H1a+Arc+cFos):HBOa	ns	0.6797
dArc-only:HBOa vs. pArc-only:HBOa	ns	0.9807
dArc-only:HBOa vs. d(H1a+cFos)-only:HBOa	ns	0.0520
dArc-only:HBOa vs. d(H1a+Arc)-only:HBOa	ns	0.0667
dArc-only:HBOa vs. d(Arc+cFos)-only:HBOa	ns	0.9178
dArc-only:HBOa vs. d(H1a+Arc+cFos):HBOa	ns	0.5444
d(H1a+cFos)-only:HBOa vs. d(H1a+Arc)-only:HBOa	ns	0.9114
d(H1a+cFos)-only:HBOa vs. d(Arc+cFos)-only:HBOa	*	**0.0450**
d(H1a+cFos)-only:HBOa vs. d(H1a+Arc+cFos):HBOa	ns	0.1787
d(H1a+Arc)-only:HBOa vs. d(Arc+cFos)-only:HBOa	ns	0.0530
d(H1a+Arc)-only:HBOa vs. d(H1a+Arc+cFos):HBOa	ns	0.2170
d(Arc+cFos)-only:HBOa vs. d(H1a+Arc+cFos):HBOa	ns	0.4782
pH1a-only:HBOa vs. pcFos-only:HBOa	****	**<0.0001**
pH1a-only:HBOa vs. pArc-only:HBOa	****	**<0.0001**
pH1a-only:HBOa vs. p(H1a+cFos)-only:HBOa	****	**<0.0001**
pH1a-only:HBOa vs. p(H1a+Arc)-only:HBOa	****	**<0.0001**
pH1a-only:HBOa vs. p(H1a+Arc+cFos):HBOa	****	**<0.0001**
pcFos-only:HBOa vs. pArc-only:HBOa	ns	0.5200
pcFos-only:HBOa vs. p(H1a+cFos)-only:HBOa	*	**0.0138**
pcFos-only:HBOa vs. p(H1a+Arc)-only:HBOa	ns	0.6283
pcFos-only:HBOa vs. p(Arc+cFos)-only:HBOa	ns	0.4411
pcFos-only:HBOa vs. p(H1a+Arc+cFos):HBOa	ns	0.5825
pArc-only:HBOa vs. p(H1a+cFos)-only:HBOa	**	0.0021
pArc-only:HBOa vs. p(H1a+Arc)-only:HBOa	ns	0.2604
pArc-only:HBOa vs. p(Arc+cFos)-only:HBOa	ns	0.8986
pArc-only:HBOa vs. p(H1a+Arc+cFos):HBOa	ns	0.2338
p(H1a+cFos)-only:HBOa vs. p(H1a+Arc)-only:HBOa	*	**0.0467**
p(H1a+cFos)-only:HBOa vs. p(Arc+cFos)-only:HBOa	**	**0.0014**
p(H1a+cFos)-only:HBOa vs. p(H1a+Arc+cFos):HBOa	ns	0.0542
p(H1a+Arc)-only:HBOa vs. p(Arc+cFos)-only:HBOa	ns	0.2106
p(H1a+Arc)-only:HBOa vs. p(H1a+Arc+cFos):HBOa	ns	0.9457
p(Arc+cFos)-only:HBOa vs. p(H1a+Arc+cFos):HBOa	ns	0.1878
d(H1a+Arc+cFos):HBOa vs. p(H1a+Arc+cFos):HBOa	ns	0.5412
d(H1a+cFos)-only:HBOa vs. p(H1a+cFos)-only:HBOa	ns	0.2313
d(H1a+Arc)-only:HBOa vs. p(H1a+Arc)-only:HBOa	ns	0.4891
d(Arc+cFos)-only:HBOa vs. p(Arc+cFos)-only:HBOa	ns	1.000

The table describes the outcome of Fisher’s multiple comparisons of immediate early gene (IEG)-positive cells in the distal CA1 (dCA1) and proximal CA1 (pCA1) regions of the hippocampus. Nuclear expression of Arc, cFos and Homer1a (H1a) (*n* = 6) were compared to expression in control hippocampus (*n* = 6). In this experiment, animals underwent only one holeboard exposure (holeboard contain objects, HBOa). In the table “only” signifies that cells were assessed that expressed only one or two of the three IEGs, a plus sign (+) indicates that double or triple labeling of nuclei was assessed. H1a expression was used to determine the effects of HBOa. Here, Arc and cFos expression were used to detect the neuronal activation at time point 40 min after novel acquisition of HBOa. The summary column reports the significance levels (*p < 0.05,

**p < 0.01,

****p < 0.0001) and indicates if effects were not significant (ns). The *P*-value column reports *p*-values, whereby significant effects are highlighted in bold font. dArc, distal CA1 Arc; dcFos, distal CA1 cFos; dH1a, distal CA1 Homer1a; pArc, proximal CA1 Arc; pcFos, proximal CA1 cFos; pH1a, proximal CA1 Homer1a; control, group of animals that did not undergo learning task; HBOa, group of animals exposed once to a novel holeboard containing novel objects; ns, not significant.

## 3 Results

### 3.1 Nuclear Homer1a expression reveals hippocampal neuronal ensembles that encode a novel spatial experience. Responses are distributed across the distal and proximal CA1

Nuclear Homer1a expression was detected as a biomarker of IEG encoding that was triggered by novel HB exposure. Nuclear Homer1a mRNA expression was significantly increased in neurons of both the distal and proximal CA1 region compared to controls [Figures 1D, E, HB (*n* = 6) vs. control (*n* = 6), mANOVA for factor “group”: *F* (1,60) = 221.993, *p* < 0.0001]. A Tukey HSD *post-hoc* test confirmed the significantly greater expression of nuclear Homer1a in the CA1 region of the HB group compared to control animals (HB vs. control *p* = 0.0001 for dCA1, and *p* = 0.0001 for pCA1). HB-induced nuclear expression of Homer1a was equivalent in dCA1 and pCA1 (Tukey HSD *post-hoc* test for HB, dCA1 vs. pCA1, *p* = 0.9050). Thus, neurons of the dCA1 and pCA1 respond equally to a novel spatial experience involving a distinct and novel spatial change to the environment.

### 3.2 Nuclear cFos expression reveals that the updating of an established spatial representation requires activation of neuronal ensembles in the distal CA1 region

The IEG cFos is involved in memory acquisition and retention, as shown by many gain-of-function and loss-of-function studies, with responses specifically evident in the CA1 region (Yelhekar et al., 2024). For this reason, we used it as biomarker for ensemble stabilization and/or expansion after novel HBO exposure. Twenty-five minutes after novel HB exposure, the animals were re-exposed to the HB, but this time novel objects were inserted into three of the four HB holes (HBO). This event was designed to prompt updating of the spatial representation, specifically to include new content information without appreciably changing the spatial environment. The HBO was inserted into the animals’ chamber 25 min after concluding HB exposure and 5 min before brain removal thereby allowing us to use nuclear cFos mRNA expression as a biomarker of neuronal activation (Saidov et al., 2018).

Here, nuclear c-Fos expression was elevated in both dCA1 and pCA1 in HBO animals compared to controls (Figures 1D, E, Tukey HSD *post-hoc* test, control vs. HB: *p* = 0.0001 for dCA1 and *p* = 0.0256 for pCA1, *n* = 6 each). This suggests that *de novo* information encoding occurred as a result of HBO exposure.

To assess whether the same, or different, neurons responded to HBO compared to HB, we examined co-labeling of H1a and cFos. Overall, a higher number of co-labeled positive neurons were evident in the CA1 region compared to controls [mANOVA (*F* (1,80) = 184.3950, *p* < 0.0001, control vs. HB: Tukey HSD *post-hoc* test for H1a+c-Fos, *p* = 0.0001 for dCA1, *p* = 0.0468 for pCA1, *n* = 6 each]. Interestingly, co-labeling effect was significantly higher in the distal CA1 compared to the proximal CA1 (HB, Tukey HSD *post-hoc* test for dCA1 vs. pCA1: *p* = 0.0495) (data not shown).

We then examined the extent to which the co-labeled population compares to the populations that expressed H1a only, or cFos only. This was done to clarify whether cFos expression resulting from HBO corresponded to an expansion of the original HB ensemble. No significant difference was detected with regard to cFos-only and H1a-only expressing neurons, in both CA1 subcompartments. Then, we examined the contribution of neurons that expressed H1a only, or cFos only, to the total number of activated neurons. Multifactorial ANOVA revealed that there was a significant difference in these populations [*F* (13,140) = 7,716, *p* < 0.0001, *n* = 6 each].

**FIGURE 2:**
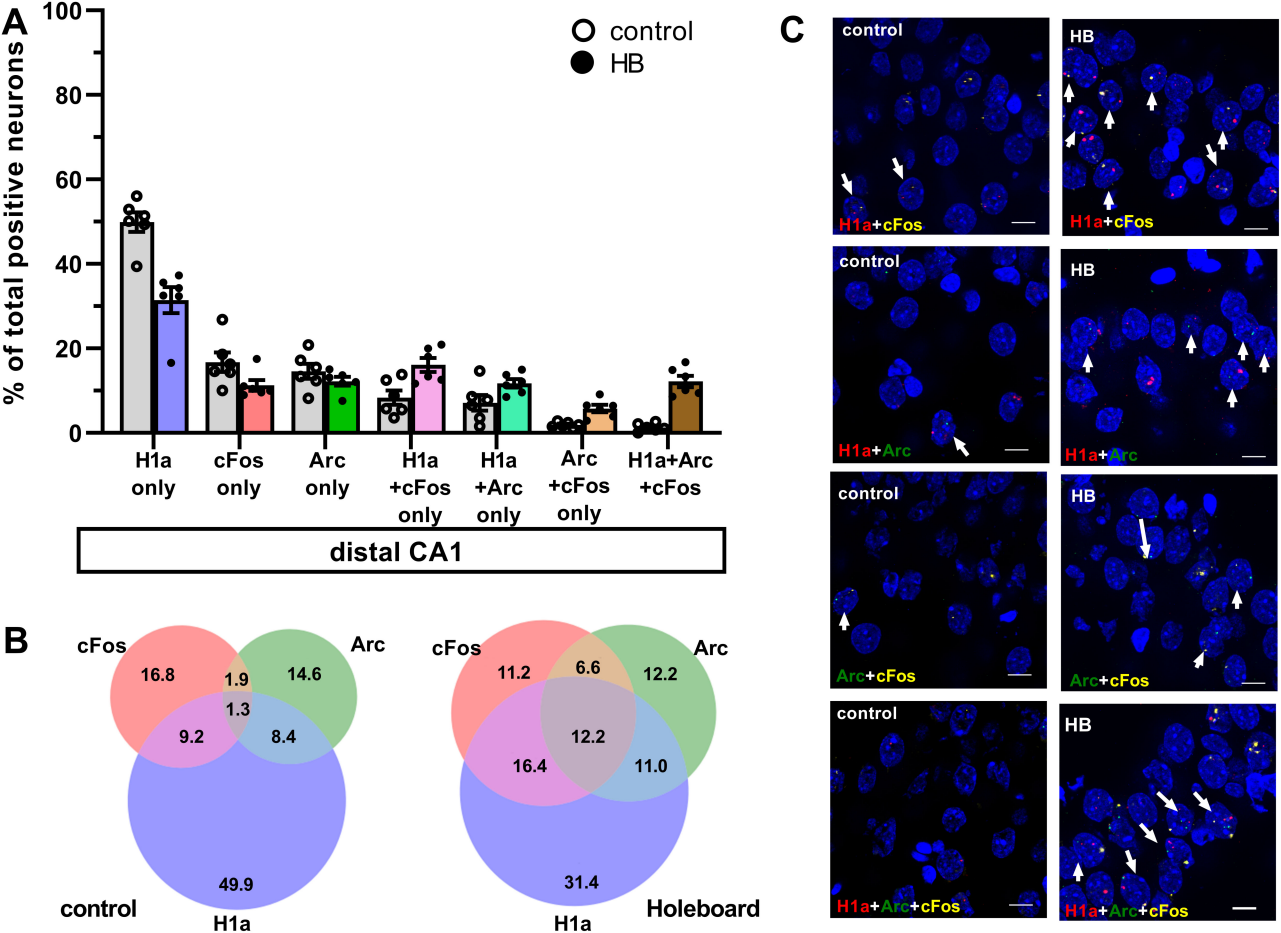
Comparison of co-labeling reveals ensemble stabilization (H1a+cFos), ensemble updating (H1a+Arc) and competitive updating (Arc+cFos and H1a+Arc+cFos) in the distal CA1. **(A)** Bar chart shows the percentage of neurons in distal CA1 (mean ± SEM) that expressed only Homer1a following novel HB exposure, or only cFos and only Arc following HBO, as well as the co-labeled populations. Percentage of dCA1 neurons that labeled Homer1a only and cFos only were significantly greater in the control group compared to the HB group. No significant differences were observed in the percentage of neurons that only expressed Arc following HBO, compared to controls. Significant effects were detected in distal CA1 when neurons that co-expressed Homer1a+cFos, Homer1a+Arc, Arc+cFos and Homer1a+Arc+cFos were compared to controls (gray). See Table 1 for statistics. Individual data points are shown as open circles for controls and filled circles for test animals (indicated as HB in chart legend). **(B)** Venn diagrams show the average percentages of each neuron population in the distal CA1 that expressed only one or co-expressed more than one IEGs. Under control condition (no exploration task), percentage of neurons that expressed only Homer1a make the most contribution among the activated neurons. The contribution of neurons that expressed c-Fos or Arc are also less compared to Homer1a. Furthermore, the contribution of co-labeling is very weak. Under learning condition (HB), the contribution shifted to the co-labeling populations. **(C)** Representative images of co-labeled neurons in the distal CA1 a rat that underwent exploration events (HB) and in a control rat (no exploration events). Nuclear Homer1a, cFos and Arc signals are indicated by red, yellow and green dots, respectively. Nuclei were counterstained with DAPI. White arrows indicate nuclei that co-expressed IEGs. Images were obtained using a wide-field fluorescence microscope at the final magnification 63×. Scale bars: 10 μm.

**FIGURE 3:**
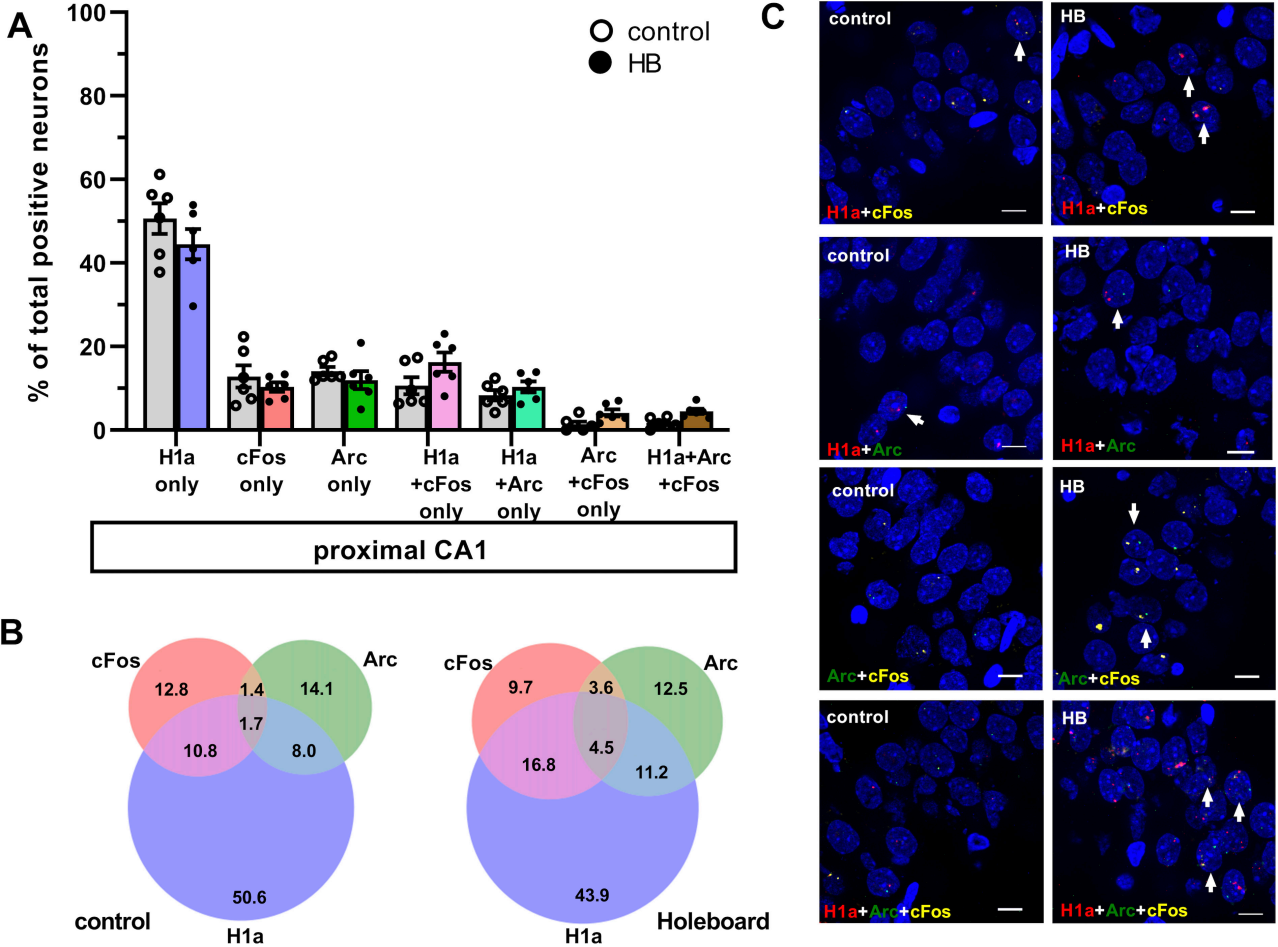
Comparison of co-labeling in the proximal CA1 reveals only ensemble stabilization but not ensemble updating. **(A)** Bar chart shows the percentage of neurons in proximal CA1 (mean ± SEM) that expressed only Homer1a following novel HB exposure, or only cFos and only Arc following HBO, as well as the co-labeling populations. Percentage of pCA1 neurons labeled H1a only was significantly greater in the control group compared to HB group. No significant differences were observed in the percentage of neurons only expressed cFos or Arc following HBO, compared to controls. Significant effects were detected in proximal CA1 when neurons that co-expressed Homer1a+cFos were compared to controls (gray). No significant differences when comparing the percentages of neurons that co-labeled Homer1a+Arc, Arc+cFos and Homer1a+Arc+cFos between HB and control groups. See Table 1 for statistics. Individual data points are shown as open circles for controls and filled circles for test animals (indicated as HB in chart legend). **(B)** Venn diagrams show the average percentages of each neuron population in the proximal CA1 that expressed only one or co-expressed more than one IEGs. Under control condition (no exploration task) percentage of neurons that expressed only Homer1a make the most contribution among the activated neurons. The contribution of neurons that expressed c-Fos or Arc are also less compared to Homer1a. Furthermore, the contribution of co-labeling is very weak. Under learning condition (HB), in proximal CA1, the effect is quite similar to the effect observed in control condition. Only the contribution of Homer1a+cFos is increased. **(C)** Representative images of co-labeled neurons in the proximalCA1 a rat that underwent exploration events (HB) and in a control rat (no exploration events). Nuclear Homer1a, cFos and Arc signals are indicated by red, yellow and green dots, respectively. Nuclei were counterstained with DAPI. White arrows indicate nuclei that co-expressed IEGs. Images were obtained using a wide-field fluorescence microscope at the final magnification 63×. Scale bars: 10 μm.

In control animals, the percentage of neurons that were co-labeled with just H1a+cFos was significantly lower than percentage of neurons that expressed H1a alone, or cFos alone. This effect was observed in both dCA1 and pCA1 (Figures 2, 3, H1a+cFos). Furthermore, there were no significant differences in the percentage of neurons that expressed Homer1a alone, or cFos alone in dCA1 compared to pCA1 (for statistical comparison see Table 1). By contrast, exposure to HB and subsequent exposure to HBO activated different populations of neurons. In dCA1, the number of neurons that expressed H1a alone was significantly greater than the population that co-labeled both H1a+cFos (Table 1). However, the proportion of solely cFos positive neurons was significantly less than the population of co-labeled neurons. In pCA1, similar effects were observed. Moreover, the percentage of H1a-only positive neurons in dCA1 was significantly less than in pCA1 (for statistical comparison, see Table 1). No significant differences in percentages of cFos alone, or co-labeled H1a+cFos neurons were observed for the comparison between dCA1 and pCA1. Our results suggest that most of the cells in the dCA1 that were activated by the first event (Homer1a), were reactivated during HBO (cFos). Neurons that were labeled by Homer1a in pCA1 following HB, were less affected by subsequent HBO.

### 3.3 Nuclear Arc expression confirms that the updating of an established spatial representation requires activation of neuronal ensembles in the distal CA1 region

To verify cFos labeling we also assessed nuclear Arc expression following HBO. Here, unlike somatic cFos expression under the same conditions, somatic Arc expression triggered by HBO was significantly increased in dCA1, but not pCA1 (Figures 1D, E, Tukey HSD *post-hoc* test, control vs. HB: *p* = 0.0001 for dCA1 and *p* = 0.0927 for pCA1, *n* = 6 each). This suggests that experience-dependent somatic expression of Arc and cFos does not occur in a homogeneous manner in hippocampal neurons.

When we assessed co-labeling of Homer1a and Arc, we observed that co-labeling was only significantly present in the dCA1 of test animals compared to controls. We also determined the percentages of neurons that expressed Arc alone or co-labeled with both Homer1a and Arc (no cFos), relative to the total activated neurons in dCA1 and pCA1 (Figures 2, 3, H1a+Arc). In control animals, the percentage of solely Arc-positive neurons was greater than percentage of neurons that exhibited co-labeling of Homer1a and Arc. These effects were evident in both dCA1 and pCA1. No significant differences were observed when comparing the percentages of these two populations between dCA1 and pCA1 in controls. By contrast, in the test group, the proportions of solely Arc positive neurons and neurons that co-labeled for only H1a+Arc were comparable in both dCA1 and pCA1. For both groups, percentage of solely H1a positive neurons was greater than the percentage of neurons that co-labeled for H1a+Arc (for statistical comparison, see Table 1).

Moreover, the proportion of neurons that co-labeled for only Homer1a and Arc was significantly less than the proportion of neurons that co-labeled for only Homer1a and cFos (Table 1).

### 3.4 Co-labeling of Arc and cFos suggests that active competition between *de novo* encoding and ensemble remodeling contributes to the updating of spatial representations

Although both Arc and cFos are required for associative learning, cFos appears to promote the recruitment of neurons into a memory representation (Yelhekar et al., 2024), whereas Arc may be involved in ensemble remodeling and the optimization/updating of established representations (Mikuni et al., 2013; Okuno et al., 2018).

To clarify if HBO resulted in different nuclear expression patterns of these IEGs, we assessed co-labeling in dCA1 and pCA1 after the learning events (Figures 2, 3, Arc+cFos). We observed that a significantly greater proportion of neurons that co-labeled for only Arc and c-Fos (no Homer1a) were detected in dCA1, compared to control animals. By contrast, pCA1 showed no significant differences between two conditions.

We then assessed how these populations compared to neurons that expressed cFos alone and Arc alone, as a result of HBO. In dCA1, the proportion of neurons that co-expressed only Arc+cFos was significantly less than the population that expressed Arc alone, but not cFos alone. In pCA1, both percentages of neurons that expressed only Arc, or only cFos were significantly less than the percentage of co-labeling. Statistical results are summarized in Table 1.

These results indicate that a competition may occur during information updating within a subpopulation of neurons that serves to determine whether the neuron becomes integrated into, or removed from, the updated neuronal ensemble. We hypothesized that those neurons that become successfully integrated into the revised ensemble may co-express all three IEGs.

### 3.5 A sparse population of neurons in the CA1 region exhibits triple Homer1a, cFos and Arc co-labeling

We detected another population of neurons in dCA1 that co-expressed all three Homer1a, Arc and cFos signals. Responses were statistically significant compared to controls. No significant differences were observed in pCA1. Responses were significantly greater in the dCA1 compared to the pCA1 (Tukey HSD *post-hoc* test, *p* = 0.02). We then examined the percentage of this neuronal population relative to the total active neurons in CA1 (Figures 2, 3, H1a+Arc+cFos). In dCA1, the population of neurons that co-labeled H1a, cFos and Arc was significantly greater than the one that co-labeled only Arc and cFos, but was less than the population that co-expressed only H1a and cFos and was comparable to the population that showed co-labeling only for H1a+Arc. Interestingly, the proportion of neurons that exhibited co-labeling of all three IEGs was comparable to that noted for neurons that expressed cFos alone and Arc alone but was significantly less compared to the percentages of neurons that expressed solely Homer1a. In pCA1, the percentage of neurons that co-expressed Homer1a, cFos and Arc was significantly less than other populations that co-expressed only H1a+cFos and only H1a+Arc, but comparable to the population that co-expressed only Arc+cFos. Furthermore, the percentage of H1a+Arc+cFos positive neurons was also significantly less than the percentage of neurons expressing H1a alone, Arc alone and cFos alone. Statistical analysis is summarized in Table 1. These results suggest that neurons of dCA1 are more engaged in information encoding and updating than neurons of pCA1.

### 3.6 Re-exposure to the spatial representation is required for the updating of memory

To address the question as to whether Arc and cFos expression is associated with updating of the HB representation, as opposed to being triggered by a random experience prior to brain removal, we additionally quantified IEG expression in another group of animals that underwent an acquisition event involving exposure to a novel holeboard that included three novel objects in the holeboard holes (HBOa). These animals did not experience a second holeboard exposure and brains were removed 40 min after the start of HBOa (Supplementary Figure 1A). A multifactorial ANOVA revealed significant effects between two conditions (control: no HB exploration versus HBOa) [*F* (1,60) = 52,31, *p* < 0.0001, *n* = 6 each]. Consistent with our previous findings, a significant increase in Homer1a expression was observed in the dCA1 region following novel acquisition to HBOa (Tukey *post-hoc* test, HBOa vs. control: *p* < 0.0001 for dCA1, and *p* = 0.1788 for pCA1) (Hoang and Manahan-Vaughan, 2024). However, no significant differences in Arc and cFos expression were observed compared to controls (Tukey *post-hoc* test, dCA1: *p* = 0.9988 for cFos, *p* = 0.9541 for Arc, pCA1: *p* = 0.8476 for cFos, *p* > 0.9999 for Arc) (Supplementary Figures 1B, C). Furthermore, a comparison of Homer1a, Arc and cFos co-labeling revealed no significant effect (Table 2 and Supplementary Figure 2). Here, only neurons that expressed H1a showed significant effects compared to controls. These results suggested that the increase in nuclear expression of Arc and cFos that we reported for HBO (Figures 2, 3) was caused by spatial experience.

## 4 Discussion

It is a common assumption that neuronal ensembles that are activated by a learning experience, comprise the repository of the acquired memory and are thus, often referred to as being “engram cells” (Frankland et al., 2024; Guan et al., 2016; Tonegawa et al., 2018). More recently however, it was reported that neurons that become activated in conjunction with the acquisition of associative fear memory, are neither static in their expression, nor function (Ko et al., 2025), and instead of reflecting a detailed memory, these cells may correspond to the retention of a gist of the original experience that can be re-utilized in subsequent learning. Moreover, studies that examined the activation and/or reactivation of neurons in non-aversive learning were determined on the basis of sole cFos, or sole Arc expression (Kwapis et al., 2019; Autore et al., 2023; Chen et al., 2022), thereby restricting insights to those neurons that express a specific IEG. Currently, little is known about the extent to which neurons, or neuronal ensembles, are re-engaged or updated during associative forms of learning such as spatial learning. Here, we report that elements of a neuronal population of the dorsal CA1 region that was recruited by novel holeboard learning, are re-activated during representational updating. Furthermore, the original ensemble changes during this information updating experience as new neurons are (sparsely) added to the ensemble. Interestingly, neurons of the proximal (pCA1) and distal CA1 (dCA1) regions were engaged in the initial ensemble selection, as well as ensemble re-activation/stabilization events (as indicated by Homer1a and cFos expression). By contrast, increases in nuclear Arc only occurred in dCA1 following exposure to the holeboard with novel objects. This suggests that neurons that are putatively activated during *de novo* spatial learning are more stable in pCA1, consistent with its putative targeting by the “where” (dorsal visual) stream, as postulated by others (Amaral and Witter, 1989; Burke et al., 2011; Hoang et al., 2018). The updating of the neuronal ensemble within the dCA1 following item insertion into the holeboard is consistent with the putative role of the dCA1 as recipient of “what” information from the ventral stream (Amaral and Witter, 1989). Moreover, this study indicates that neurons, that are initially recruited during novel spatial learning, form a scaffold that is subsequently modified and updated during integration of further details of this representation.

In this study, we used a multiplexed FISH approach to differentiate neurons of the hippocampal dorsal CA1 region that engaged in nuclear IEG expression, as a result of novel holeboard exposure, from those that expressed IEG due to the updating of this environment by the inclusion of objects in three of the four holeboard holes. Previous studies have shown that these two behavioral learning events are tightly associated with the expression of hippocampal synaptic plasticity in the form of persistent LTP and LTD, respectively (Hagena and Manahan-Vaughan, 2024). We focused specifically on nuclear IEG expression in pCA1 and dCA1 because of their abovementioned putative functions in the processing of item-related (what) and location-related (where) elements of spatial information processing. We were particularly interested in the stability of neurons that were activated by the first learning experience (novel holeboard exposure), given that in the timeframe of the experiment (40 min), consolidation is unlikely to have occurred (Inostroza et al., 2013). We used Homer1a as a biomarker of neuronal activation during *de novo* holeboard learning, and timed brain removal for FISH assessments to align with the peak nuclear expression of Homer1a after initiation of this experience (35–40 min), (Hoang et al., 2018, 2021). The specificity of this timing was validated by the very low level of cytoplasmic Homer1a staining in our preparations (Figures 1, 2). Animals were well-habituated to the experimenter and test chamber and were kept in a quiet environment prior to experiments. An extensive cytoplasmic signal in the brain slices would reflect prior experience-dependent induction of Homer1a expression, that in our experimental environment could only have been induced by stress. Moreover, the very low cytoplasmic signal confirms that peak nuclear expression of Homer1a occurred 35–40 min after novel holeboard exposure.

We observed that neurons of both the pCA1 and dCA1 showed significant increases in nuclear expression of Homer1, as a result of novel holeboard exposure. Previously, we have reported that the facilitation of hippocampal LTP in the CA1 region of freely behaving rats, by novel holeboard exposure, results in increased nuclear Homer1a expression in the dCA1 and pCA1 (Hoang et al., 2021), as does exposure to a novel holeboard (HB) in the absence of electrophysiological stimulation (Hoang et al., 2018). Effects are consistent with the encoding of both “what” and “where” elements of this spatial experience. We used nuclear Arc and cFos expression as biomarkers of neuronal activation that occurred when items were inserted in the holeboard holes (HBO), this event occurred 25 min after novel holeboard exposure and 5 min prior to brain removal, thereby allowing us to compare IEG responses with Homer1a expression. Peak nuclear expression of Homer1a occurs ca. 40 min after a specific experience, whereas peak nuclear nuclear Arc and cFos expression occurs 5–6 min and 6–8 min after the experience, respectively (Hoang et al., 2018; Saidov et al., 2018; Vazdarjanova et al., 2002). The timing of the experiment (5 min of HBO prior to brain removal), plus the time needed for brain removal, will have meant that we were in the range of peak nuclear expression of cFos, as well as Arc. Interestingly, as was the case for Homer1a, nuclear cFos expression was increased in both dCA1 and pCA1 following HB, compared to controls.

Whereas novel exposure of rats to a HB facilitates the expression of hippocampal LTP, novel HBO facilitates LTD in the CA1 region (Kemp and Manahan-Vaughan, 2004, 2008b; Manahan-Vaughan and Braunewell, 1999). Both plasticity forms are tightly associated with spatial learning and information updating (Hagena and Manahan-Vaughan, 2024). Notably, the inhibition of learning in rodents, by means of pharmacological antagonism of aminergic receptors (Kemp and Manahan-Vaughan, 2004, 2008a), antagonism of glutamate receptors (Popkirov and Manahan-Vaughan, 2011; Goh and Manahan-Vaughan, 2013), or optogenetic control of catecholamine release in the hippocampus, also prevents the synaptic plasticity response (Kemp and Manahan-Vaughan, 2004; Hagena and Manahan-Vaughan, 2024, 2025). Furthermore, intracerebral application of a c-fos antisense oligonucleotide in rats prevents LTD that results from HBO (Kemp et al., 2013). This indicates that both novel holeboard learning, and novel learning of the item constellations in the holeboard holes, took place in the paradigm used in the current study, and that IEG expression was triggered by these experiences. In line with this interpretation, no significant increases in Arc or cFos expression occurred if the second learning event did not occur, and brains were removed 40 min after single exposure of the animals to a novel holeboard containing novel objects. In other words, Arc and cFos expression were triggered by HBO exposure.

The increase in nuclear Arc and cFos expression after HBO exposure raised the question as to whether this reflected the recruitment of additional neurons into the HB ensemble, or if the increased IEG expression corresponded to the activation of same neurons that were activated by the *de novo* holeboard exposure. For this we looked for double labeling of nuclei with cFos and Homer1a. Strikingly, the number of neurons that expressed only cFos, or only Homer1a, was not increased compared to controls. By contrast, the population that co-expressed cFos and Homer1a was significantly greater in both distal and proximal CA1. However, significantly more neurons co-expressed Homer1a and cFos in dCA1 compared to pCA1. This suggests that when animals were re-exposed to the now familiar holeboard that contained novel objects (HBO), the same neurons that were activated during novel holeboard exposure were once more activated during the updating of the holeboard information, especially with regard to “what” information. This finding contradicts reports by others that neuronal cFos expression that is triggered by fear learning typically overlaps with other IEG-expressing neuronal ensembles (Vazdarjanova and Guzowski, 2004) and rather suggests that the use of benign spatial learning paradigms may reveal nuances of experience encoding that are not possible when fear learning is implemented.

Another possible interpretation of the double-labeling of nuclei with Homer1a and cFos is that this reflected memory retrieval. Retrieval after memory consolidation is unlikely for the following reasons: Memory consolidation in rats requires longer than ca. 25 min (Inostroza et al., 2013). Axonal transport of proteins from the nucleus to the synapse takes between 0.2 and 200 mm/day depending on the structure of the protein (Roy, 2020), meaning the fastest transport will take >1 h, depending on the length of the axon. Homer1a modulates synaptic function, especially through interactions with metabotropic glutamate receptor-5 (mGlu5) (Ango et al., 2000; Chokshi et al., 2019). Given the above-mentioned rates of axonal transport, it is unlikely however, that 35 min after novel holeboard exposure, new proteins, transcribed as a result of nuclear Homer1a expression, would have reached the synapse. Thus, the cFos signal that we detected in neurons, that also expressed Homer1a, is unlikely to reflect a memory retrieval response, subsequent to memory consolidation, and is much more likely to reflect the consistent activation of the same neuron in the novel holeboard and HBO updating events. The double labeling of neurons with Homer1a and cFos can, of course, arguably correspond to retrieval of short-term memory, although studies that have addressed the possibility of hippocampal and CA1 involvement in working, or short-term memory retrieval, used longer timelines than our paradigm (Yoshihara and Ichitani, 2004; Duda and Wesierska, 2021; see also: Lee and Kesner, 2002). That being said, re-exposure of rodents to a familiar environment results in a reiteration of IEG expression in neurons of the dentate gyrus that were previously activated by novel environment exposure (Gulmez Karaca et al., 2021). To clarify whether similar effects occur in CA1, experiments would be needed that repeat the exact same conditions twice (e.g., HB-HB, HBO-HBO).

Activity-regulated cytoskeleton-associated protein plays a specific role in the retention of long-term memory (Attardo et al., 2018; Plath et al., 2006), as well as persistent forms of hippocampal LTP and LTD (Hoang et al., 2021; Messaoudi et al., 2007; Shepherd and Bear, 2011). It has been ascribed a role in the maintenance of changes of synaptic strength within physiological ranges (synaptic scaling) (Park et al., 2008), the spatial distribution of synaptic weights (Okuno et al., 2018), and in the elimination of synapses (Mikuni et al., 2013; Wilkerson et al., 2018). Given that Arc has been reported to support behavioral tagging (Moncada et al., 2011), its role in synaptic scaling may occur by a process that involves Arc accumulation at activated synapses, whereas weakened synapses experience activity-dependent Arc mRNA degradation (Farris et al., 2014) and AMPA receptor endocytosis (Park et al., 2008; Plath et al., 2006). Based on these findings, we interpreted nuclear Arc signals as indicating that a neuron may be competitively undergoing synaptic scaling associated with HBO. Consistent with this interpretation we did not detect an increase in the percentage of neurons that were labeled with Arc alone, compared to controls. However, we detected a significant population of neurons in dCA1 (and not pCA1) that co-labeled for Homer1a and Arc. We cannot currently exclude the possibility that Arc expression is less sensitive in response to spatial experience than Homer1a and cFos, and that this was why pCA1 expression of Arc in Homer1a-positive cells was weak. However, given that dCA1 is believed to process “what” aspects of information, the co-labeling of neurons with Homer1a and Arc could on the one hand reflect the updating of the neuronal ensemble with novel item information, and on the other hand, indicate that co-labeled neurons will be eliminated from the original dCA1 ensemble. If the latter possibility is valid, we should also see a significant percentage of neurons that co-label with cFos and Arc in dCA1. This was indeed the case. A similar significant outcome was obtained when we assessed the percentage of neurons that co-expressed Homer1a, cFos and Arc. Thus, these neurons may have been subsequently removed from the ensemble or may have been engaged in a competitive process whereby, their synaptic weights were adjusted in a spine-specific manner.

In summary, we show in this study that a neuronal ensemble is activated in the cornus ammonis by *de novo* spatial learning that is distributed across the dCA1 and pCA1 (as reflected by nuclear Homer1a expression), consistent with the integration of information about “what” and “where” aspects of space, respectively. The subsequent introduction of novel objects into the holeboard holes prompted a re-activation of the previously activated dCA1 ensemble (as reflected by co-labeling of nuclei with Homer1a and cFos), presumably related to the integration of new “what” information in the holeboard representation, and re-exposure to the now familiar holeboard. This process was also accompanied by the expression of Arc in the dCA1 of the previously activated ensemble, consistent with an experience-dependent modification of the neuronal ensemble triggered by updating of the holeboard representation. These findings suggest that experience-dependent nuclear IEG expression is not an homogenous phenomenon and that different neurons within an ensemble may encode different information. Moreover, Homer1a may be a useful biomarker of cells that stably retain encoded information, whereas Arc may serve as a biomarker of experience-dependent changes in ensemble dynamics.

## Data Availability

The raw data supporting the conclusions of this article will be made available by the authors, without undue reservation.

## Supplementary material

Supplementary Figure 1 Exposure to novel spatial environment significantly increases nuclear expression of Homer1a in the CA1 region. **(A)** Experimental design: After having resided in the same experiment room overnight, animals underwent 1 h-habituation in the test chamber on the day of the experiment. During the first event, a holeboard containing 3 small objects was introduced to the animal in the chamber. After 10 min exploration time, the holeboard was removed. Animals rested in the chamber for 30 min until brain extraction. **(B)** Bar chart shows the percentage (mean ± SEM) of total exploration time for the exposure to holeboard with objects (HBOa). Animals spent most of the time exploring the novel spatial environment. **(C)** Bar charts show the relative percentage (mean ± SEM) of nuclear Homer1a (red bars), cFos (yellow bars) and Arc (green bars) mRNA expression in neuronal nuclei of the distal (left) and proximal CA1 (right). Novel acquisition of a holeboard containing objects (HBOa) led to a significant increase in Homer1a expression in distal CA1 compared to controls (no exploration event). No significant differences in cFos and Arc expression were observed in the CA1 of two groups (****p* < 0.001). **(D)** Representative images of nuclear Homer1a, cFos and Arc mRNA expression in the distal CA1 of a rat that underwent exploration tasks (HBOa, bottom row) and in a control rat (control, no exploration event, upper row). Nuclear Homer1a, cFos and Arc mRNA signals are indicated by red, yellow and green dots, respectively. Nuclei were counterstained with DAPI. White arrows indicate IEG positive nuclei. Images were acquired using a wide-field fluorescence microscope at the final magnification of 63×. Scale bars: 20 μm.

Supplementary Figure 2 Comparison of co-labeling reveals no evidence for ensemble stabilization (H1a+cFos), ensemble updating (H1a+Arc) and competitive updating (Arc+cFos and H1a+Arc+cFos) when the re-exposure event is absent. **(A)** Bar chart shows the percentage of neurons in distal CA1 (mean ± SEM) that expressed only Homer1a following novel HBOa exposure, or only cFos and only Arc as well as the co-labeling populations. No significant differences were observed in the percentage of neurons only expressed Homer1a, Arc and cFos, compared to controls. For both groups, percentage of dCA1 neurons that labeled Homer1a only was significantly greater compared to other populations. When the re-exposure did not occur, no significant differences were observed in the percentage of neurons only expressed Arc and cFos, compared to controls. No significant effects were detected for the co-labeling populations in distal CA1 of HBOa and control groups. See Table 2 for statistics. Individual data points are shown as open circles for controls and filled circles for test animals (indicated as HB in chart legend). **(B,E)** Venn diagrams show the average percentages of each neuron population in the distal CA1 **(B)** and proximal CA1 **(E)** that expressed only one or co-expressed more than one IEGs. No differences in the contribution of each neuron population to the total number of activated neurons were detected when comparing between learning (HBOa) and control condition. **(D)** Bar chart shows the percentage of neurons in proximal CA1 (mean ± SEM) that expressed only Homer1a following novel HBOa exposure, or only cFos and only Arc as well as the co-labeling populations. Similar to the effects observed in dCA1, no significant differences were observed in the percentage of neurons only expressed Homer1a, Arc and cFos, compared to controls. For both groups, percentage of pCA1 neurons that labeled Homer1a only was significantly greater compared to other populations. No significant effects were detected for the co-labeling populations in proximal CA1 of HBOa and control groups. See Table 2 for statistics. Individual data points are shown as open circles for controls and filled circles for test animals (indicated as HB in chart legend). **(C,F)** Representative images of co-labeled neurons in the distal CA1 **(C)** and proximal CA1 **(F)** of a rat that underwent exploration events (HBOa) and in a control rat (no exploration events). Nuclear Homer1a, cFos and Arc signals are indicated by red, yellow and green dots, respectively. Nuclei were counterstained with DAPI. White arrows indicate nuclei that co-expressed IEGs. Images were obtained using a wide-field fluorescence microscope at the final magnification 63×. Scale bars: 20 μm.
