# Supplementary figures and images for: Multiplexed detection of nuclear immediate early gene expression reveals hippocampal neuronal subpopulations that engage in the acquisition and updating of spatial experience
Source: Front Integr Neurosci. 2025 Dec 3;19:1660536. doi: 10.3389/fnint.2025.1660536 (PMC12708613; doi:10.3389/fnint.2025.1660536)

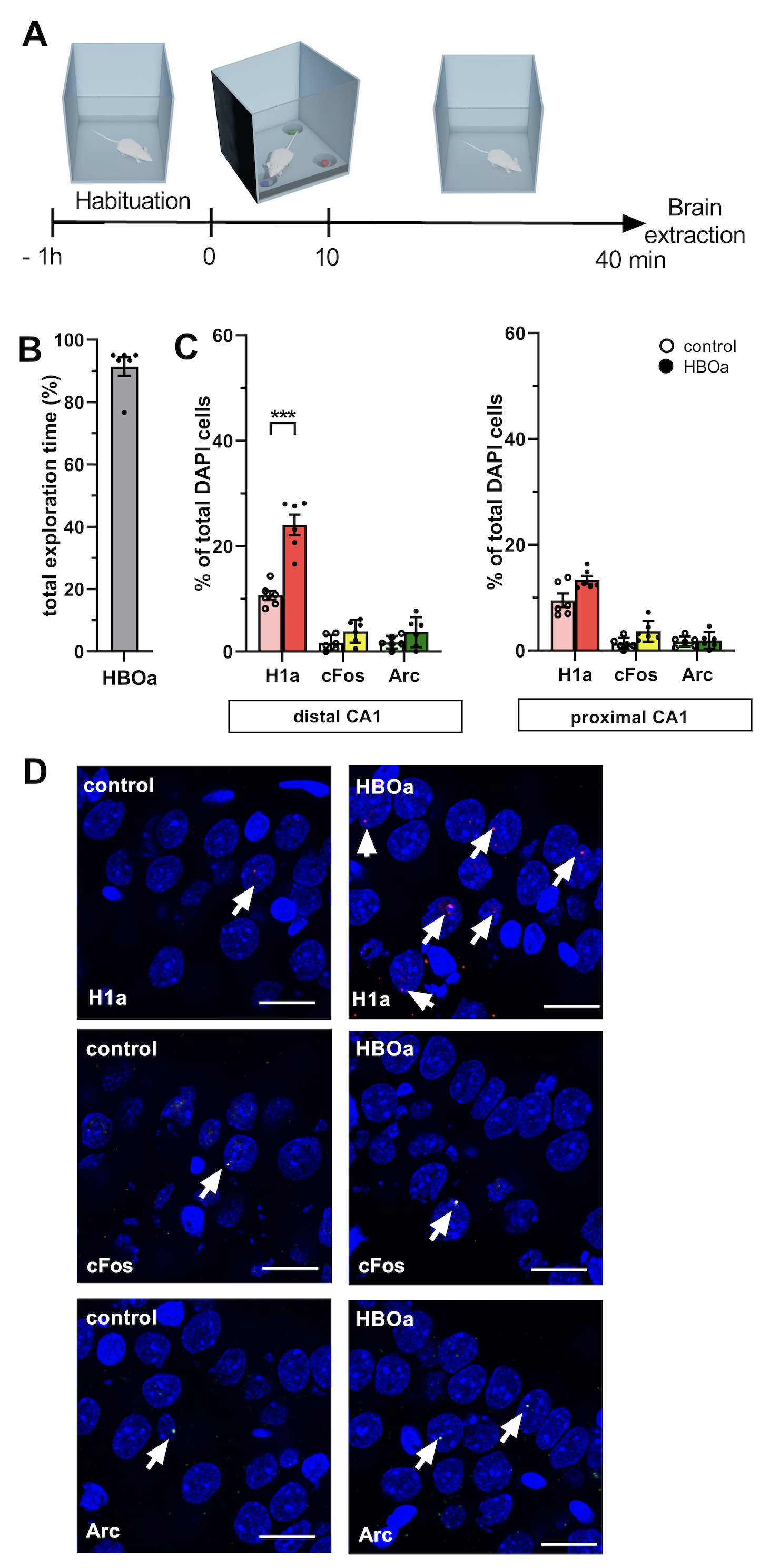

Supplement: Supplementary Figure 1 — Exposure to novel spatial environment significantly increases nuclear expression of Homer1a in the CA1 region. (A) Experimental design: After having resided in the same experiment room overnight, animals underwent 1 h-habituation in the test chamber on the day of the experiment. During the first event, a holeboard containing 3 small objects was introduced to the animal in the chamber. After 10 min exploration time, the holeboard was removed. Animals rested in the chamber for 30 min until brain extraction. (B) Bar chart shows the percentage (mean ± SEM) of total exploration time for the exposure to holeboard with objects (HBOa). Animals spent most of the time exploring the novel spatial environment. (C) Bar charts show the relative percentage (mean ± SEM) of nuclear Homer1a (red bars), cFos (yellow bars) and Arc (green bars) mRNA expression in neuronal nuclei of the distal (left) and proximal CA1 (right). Novel acquisition of a holeboard containing objects (HBOa) led to a significant increase in Homer1a expression in distal CA1 compared to controls (no exploration event). No significant differences in cFos and Arc expression were observed in the CA1 of two groups (***p < 0.001). (D) Representative images of nuclear Homer1a, cFos and Arc mRNA expression in the distal CA1 of a rat that underwent exploration tasks (HBOa, bottom row) and in a control rat (control, no exploration event, upper row). Nuclear Homer1a, cFos and Arc mRNA signals are indicated by red, yellow and green dots, respectively. Nuclei were counterstained with DAPI. White arrows indicate IEG positive nuclei. Images were acquired using a wide-field fluorescence microscope at the final magnification of 63×. Scale bars: 20 μm. [file Image_1.tif]

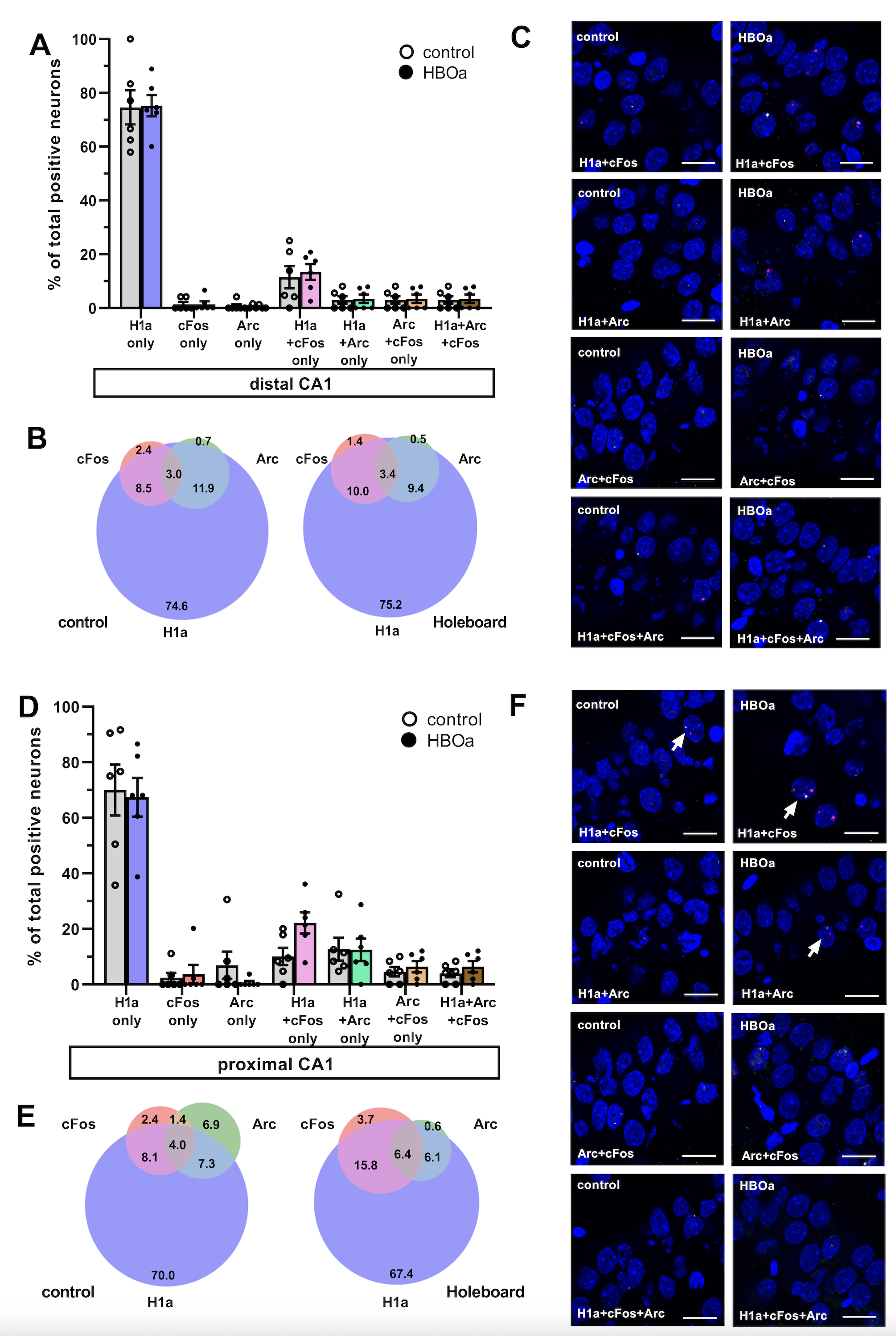

Supplement: Supplementary Figure 2 — Comparison of co-labeling reveals no evidence for ensemble stabilization (H1a+cFos), ensemble updating (H1a+Arc) and competitive updating (Arc+cFos and H1a+Arc+cFos) when the re-exposure event is absent. (A) Bar chart shows the percentage of neurons in distal CA1 (mean ± SEM) that expressed only Homer1a following novel HBOa exposure, or only cFos and only Arc as well as the co-labeling populations. No significant differences were observed in the percentage of neurons only expressed Homer1a, Arc and cFos, compared to controls. For both groups, percentage of dCA1 neurons that labeled Homer1a only was significantly greater compared to other populations. When the re-exposure did not occur, no significant differences were observed in the percentage of neurons only expressed Arc and cFos, compared to controls. No significant effects were detected for the co-labeling populations in distal CA1 of HBOa and control groups. See Table 2 for statistics. Individual data points are shown as open circles for controls and filled circles for test animals (indicated as HB in chart legend). (B,E) Venn diagrams show the average percentages of each neuron population in the distal CA1 (B) and proximal CA1 (E) that expressed only one or co-expressed more than one IEGs. No differences in the contribution of each neuron population to the total number of activated neurons were detected when comparing between learning (HBOa) and control condition. (D) Bar chart shows the percentage of neurons in proximal CA1 (mean ± SEM) that expressed only Homer1a following novel HBOa exposure, or only cFos and only Arc as well as the co-labeling populations. Similar to the effects observed in dCA1, no significant differences were observed in the percentage of neurons only expressed Homer1a, Arc and cFos, compared to controls. For both groups, percentage of pCA1 neurons that labeled Homer1a only was significantly greater compared to other populations. No significant effects were detected for the co-labeling p [file Image_2.tif]
